# Diffuse Large B-Cell Lymphoma of the Colon in an Asymptomatic Patient

**DOI:** 10.7759/cureus.26003

**Published:** 2022-06-16

**Authors:** Sai Samyuktha Bandaru, Vishal Busa, Sanjay Juneja

**Affiliations:** 1 Internal Medicine, Baton Rouge General Medical Center, Baton Rouge, USA; 2 Hematology and Medical Oncology, Mary Bird Perkins Cancer Center, Baton Rouge, USA

**Keywords:** histopathology (hp), chemotherapy, colon, diffuse large b cell lymphoma, lymphoma

## Abstract

Extranodal lymphomas of the gastrointestinal (GI) tract are known entities, but primary lymphoma of the colon is extremely rare. Symptoms are non-specific, such as abdominal pain, bloody diarrhea, unintentional weight loss, night sweats, and changes in bowel habits. Some patients do not have any specific symptoms, which makes diagnosis extremely difficult. We present a 69-year-old asymptomatic male who was incidentally found to have an inflammatory mass in the descending colon on screening colonoscopy; the initial biopsy was inconclusive. However, due to high suspicion of any underlying malignancy, a repeat colonoscopy with biopsy was done, which revealed diffuse large B-cell lymphoma (DLBCL). Prompt and early diagnosis is extremely crucial for timely management. Management includes chemotherapy, radiotherapy, and surgery.

## Introduction

Non-Hodgkin's lymphoma is a lymphoproliferative disorder originating from B lymphocytes or T lymphocytes or natural killer cells. Diffuse large B-cell lymphoma (DLBCL) is the most common subtype of non-Hodgkin's lymphoma. Primary diffuse large B-cell lymphoma of the colon is extremely rare. It affects both men and women, with male predominance and the peak incidence seen in the sixth to seventh decades [1,2]. There are only a few cases of DLBCL of the colon reported so far. Histopathological diagnosis can confirm DLBCL. Treatment modalities include chemoradiotherapy; however, the role of surgery in the treatment of DLBCL is still controversial. Some authors believe surgery should be limited to obstruction, perforation, and hemorrhage, while others believe it should be an initial treatment modality [2,3].

## Case presentation

A 69-year-old Caucasian male presented to a gastroenterology clinic for a routine screening colonoscopy. He had a two-year history of night sweats; otherwise, he did not have any symptoms. He denied unintentional weight loss, fatigue, and change in bowel habits; abdominal pain; nausea; and vomiting. The medical history was significant for hypertension, dyslipidemia, and coronary artery disease. Surgical history was significant for percutaneous coronary intervention for coronary artery disease and hernia repair. He had a 20-year history of smoking and no alcohol or drug use. No significant family history. On physical examination, vitals were stable. The lungs were clear on examination, the heart sounds were regular in rate and rhythm, there was no abdominal tenderness, bowel sounds were normal, and no frank blood was seen on rectal examination. The rest of the physical examination was unremarkable.

**Figure 1:**
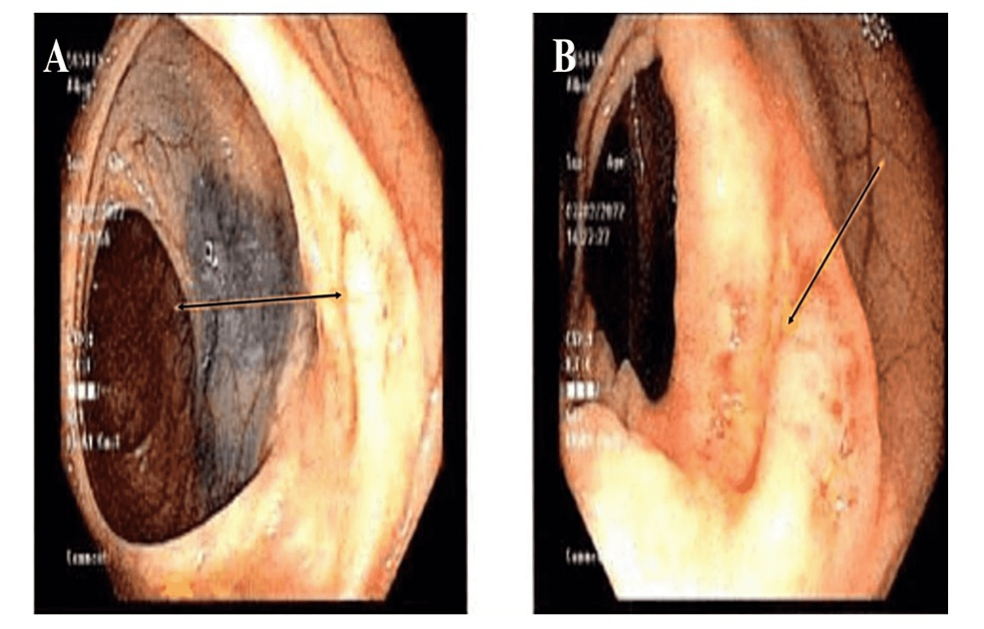
Image showing localized inflammation in descending colon (arrows in panel A, B).

**Figure 2:**
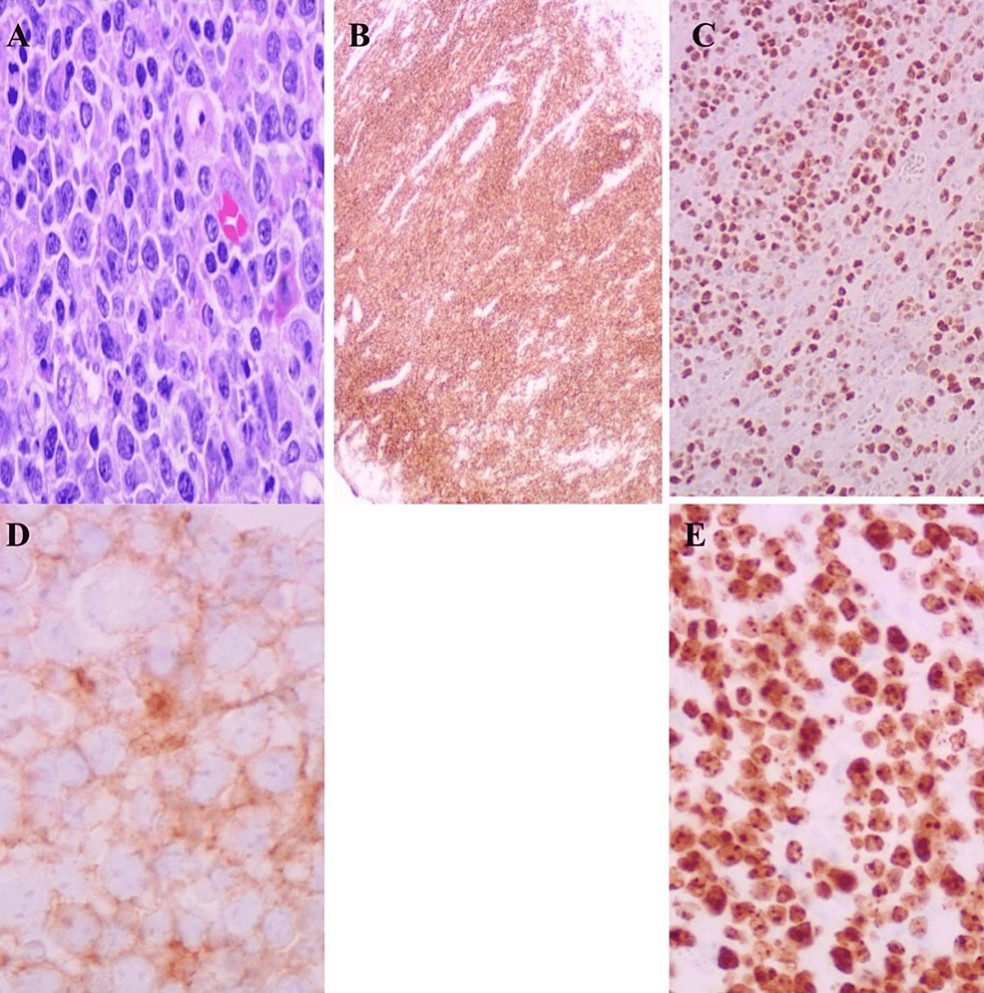
Image shows pathological confirmation of DLBCL with immunofixation staining (panels A, B, C, D, E). Panel B positive for CD20, panel C positive for BCL-6, panel D positive for CD10, and panel E positive for Ki-67 high proliferation rate.

Initial screening colonoscopy showed a 4 cm ulcerated, non-bleeding, non-obstructing mass in the descending colon (Figure 1). A biopsy of the descending colon mass showed ulcerated mucosa with fibrino-purulent exudate and granulation tissue, negative for any dysplasia or malignancy. Immunostains for cytokeratin, synaptophysin, and CDX2 were negative for evidence of invasive carcinoma or neuroendocrine tumor. However, due to high suspicion of any underlying malignancy, a repeat colonoscopy was done three months later, which revealed localized inflammation characterized by congestion, erosions, and erythema in the descending colon, and biopsies were taken for further histological examination. A biopsy from a descending colon lesion showed high-grade CD10-positive B-cell lymphoma with intermediate to large-sized lymphoid cells. Immunohistochemistry staining was positive for CD20, CD10, and BCL6, but negative for CD 30 (Figure 2). Fluorescence in situ hybridization (FISH) analysis did not reveal any abnormalities in BCL2, BCL6, or MYC. The Ki-67 proliferative index is high (around 80 to 90%). The initial staging positron emission tomography (PET) scan showed no evidence of 18F-fluorodeoxyglucose (FDG) avid disease. Based on pathology and PET scan findings, he was diagnosed with diffuse large B-cell lymphoma stage I of the descending colon. He established care with a hematologist for further management.

He underwent a laparoscopic colon resection. 20.1 cm of splenic flexure and proximal descending colon segment were resected; the serosa of the resected lesion was pink- tan and smooth; multiple possible lymph nodes were resected and sent for immunohistological examination. The histology of the resected colon revealed a 1.4 cm atypical lymphoid lesion involving mucosal, submucosal, and superficial midmuscularis propria; it is composed of intermediate to large lymphocytes with diffuse pattern involvement. Immunohistochemistry was positive for CD10, CD20, BCL6, and negative for CD30 and MUM1. The proliferative index Ki-67 is high, around 95%. Morphological and immunophenotypic findings are consistent with diffuse large B-cell lymphoma. Negative FISH for MYC and large cell size make Burkitt lymphoma unlikely. All the resected lymph nodes were negative for any metastatic disease. He was seen by a hematologist and was started on chemotherapy with six cycles of rituximab, cyclophosphamide, hydroxydaunorubicin, oncovin, and prednisone (R-CHOP). He was seen after two cycles of R-CHOP. He is tolerating chemotherapy well without any dose-limiting toxicities.

## Discussion

DLBCL is one of the aggressive lymphomas that can be cured, even in advanced cases, with effective treatment regimens. It is the most common lymphoma in adults, accounting for 37% of B-cell lymphomas and 30-40% of non-Hodgkin's lymphomas [4]. These commonly originate from lymph nodes, spleen, and thymus, which are grouped as nodal DLBCL. However, 30-40% of patients could be extra-nodal in origin, which includes sites like the gastro-intestinal tract, skin and soft tissue, head and neck, nervous system, genitourinary, breast, musculoskeletal, pancreaticobiliary, respiratory tract, etc. [4,5]. Among these extra-nodal sites, the GI tract is the most commonly involved site, especially the stomach in 65%, the small intestine in 20-35%, and the colon and rectum in 5-15% [6]. Typically, these patients present with gastrointestinal (GI) symptoms like abdominal pain, nausea, vomiting, weight loss, diarrhea, and rarely, can present as acute complete or partial bowel obstructions. Presentations of these patients vary widely based on the site involved. Although B symptoms are common in nodal DLBCL, the majority of patients with colonic DLBCL might be asymptomatic or with mild GI symptoms like in our patient, which was an incidental finding on screening colonoscopy. Given the extra-nodal origin, the patient might not have swollen or enlarged lymph nodes, which is a common presentation in nodal lymphomas.

Risk factors are mostly similar to nodal DLBCL with some differences, like associations with inflammatory bowel disease, celiac disease, and infections like *Campylobacter jejuni* and *Helicobacter pylori*. Other risk factors include viral infections like HIV, Epstein-Barr virus (EBV), hepatitis C virus (HCV), human T-cell leukemia virus-1 (HTLV-1), and human herpesvirus-8 (HHV-8); immunosuppressants and immunodeficiencies [7]. Pathogenesis includes activation of protooncogenes, chromosomal mutations by various mechanisms like translocation t (14;18), c-rel amplification, histone methyl-transferase mutations, and PTEN deletion [8,9]. Among these translocations, t (14;18) is the most common one seen in 30-40% of cases that leads to BCL2 overexpression, resulting in unchecked growth of B-cells in germinal centers [9].

Diagnosing colonic lymphomas needs imaging modalities like contrast-enhanced CT of the abdomen along with double-contrast barium enema followed by colonoscopy and biopsy for confirmation, as imaging only provides information about size, depth of invasion, and nodal involvement. They also needed a wide range of hematological testing and further staging of lymphoma, along with prognostication of the type of DLBCL. Basic labs include complete blood count (CBC) with a differential that helps to find the bone marrow involvement; a peripheral smear, comprehensive metabolic panel (CMP), serum lactate dehydrogenase (LDH), and uric acid levels usually correspond to tumor burden and are also used as an early marker for relapse [10]. Testing for hepatitis B virus (HBV), HCV, HIV, and EBV is necessary as these are highly lymphoproliferative viruses and these can be possible etiologies. These serologies are not only needed in diagnosis but also needed before starting treatment as they tell you the need for anti-viral prophylaxis to prevent re-activation [11,12]. Although biopsy confirms the diagnosis, further comprehensive testing like immunohistochemistry and flow cytometry needs to be done in order to identify the molecular and phenotypic variants [12]. Immunophenotyping is almost mandatory in all DLBCL cases as this confirms the B-cell lineage and type of DLBCL. The World Health Organization (WHO) has suggested an immunohistochemical panel for phenotyping that includes CD20, CD79a, BCL6, CD10, MYC, BCL2, Ki67, IRF4, CyclinD1, CD5, CD23, and EBER-1 staining for EBV [10]. Flow cytometry and cytogenetic tests like fluorescent in situ hybridization (FISH) analysis are also part of investigations that are used especially to differentiate the subtypes of DLBCL [12]. FISH studies help in identifying translocations, amplifications, and deletions, which can further guide the therapies. Imaging studies include computed tomography (CT) of the neck, chest, abdomen, and pelvis as a routine standard of care along with a whole-body PET as it helps in prognostication and staging [10]. Staging is important in any malignancy as it helps in determining the treatment plans, predicting the outcomes and survival rates, and helps in the aggressiveness of the management. Even in colonic lymphomas, the Ann-Arbor staging system is the standard classification system widely used worldwide [13].

Treatment modalities include multi-agent chemotherapy, surgical resection, and radiotherapy, and these are used alone or in combination based on the staging of lymphoma, subtype, and other co-morbidities that vary between individuals. Even in colonic lymphomas, R-CHOP induction therapy is the gold standard regimen that includes rituximab in combination with cyclophosphamide, doxorubicin (hydroxydaunorubicin), vincristine (Oncovin), and prednisone [14]. The role of surgery is still debated, and studies recommend that it should be reserved for patients with complications like obstruction, perforation, and bleeding [14]. Evidence has shown improved survival with chemotherapy +/- radiotherapy alone. In early stages and localized colonic lymphomas, a few studies showed a better outcome with surgical resection followed by chemotherapy [7]. In advanced stages, anthracycline-based multiagent chemotherapy with or without radiotherapy is indicated [7]. Typically, each cycle lasts for 14-21 days, and the number of cycles of chemotherapy is based on staging, prognosis, and need for radiation. Remission is achieved in two-thirds of patients with this regimen. Management of relapse and refractory diseases depends on whether the patient is a candidate for a hematopoietic stem cell transplant. High-dose chemotherapy with autologous stem cell rescue is shown to improve survival in these patients [1]. The overall prognosis of colonic lymphomas is poor, and these patients need to be monitored at regular intervals with frequencies based on response to initial chemotherapy and staging of the disease. Follow-ups are recommended every three to six months for three to five years, then annually or as clinically indicated with basic blood workup and LDH during each visit [4].

Our patient was completely asymptomatic, and his lymphoma was identified on routine screening colonoscopy. This case helps us realize that DLBCL of the colon can be asymptomatic. Any concerning lesions on colonoscopy need proper histopathological examination. Without an appropriate and timely diagnosis, the prognosis is poor.

## Conclusions

DLBCL of the colon is an extremely rare entity. Patients can be asymptomatic or have non-specific symptoms that can delay diagnosis. Histopathological examination findings are key for diagnosis. Early diagnosis is really important for the timely management of these patients. Treatment includes chemotherapy and radiotherapy, and the role of surgery is still controversial.

## References

[1] Erginoz E, Askar A, Cavus GH, Velidedeoglu M (2021). Primary diffuse large B-cell lymphoma of the sigmoid colon. Int J Surg Case Rep.

[2] Pandey M, Swain J, Iyer HM, Shukla M (2019). Primary lymphoma of the colon: report of two cases and review of literature. World J Surg Oncol.

[3] Haddad I, El Kurdi B, El Iskandarani M, Babar S, Young M (2019). Primary diffuse large B-cell lymphoma of the sigmoid colon. Cureus.

[4] Zelenetz AD, Gordon LI, Chang JE (2021). NCCN Guidelines® insights: B-cell lymphomas, version 5.2021. J Natl Compr Canc Netw.

[5] Vitolo U, Seymour JF, Martelli M (2016). Extranodal diffuse large B-cell lymphoma (DLBCL) and primary mediastinal B-cell lymphoma: ESMO Clinical Practice Guidelines for diagnosis, treatment and follow-up. Ann Oncol.

[6] Gupta V, Singh V, Bajwa R (2022). Site-specific survival of extra nodal diffuse large B-cell lymphoma and comparison with gastrointestinal diffuse large B-cell lymphoma. J Hematol.

[7] Boussios S, Zerdes I, Vassou A (2018). Extranodal diffuse large B-cell lymphomas: a retrospective case series and review of the literature. Hematol Rep.

[8] Martelli M, Ferreri AJ, Agostinelli C, Di Rocco A, Pfreundschuh M, Pileri SA (2013). Diffuse large B-cell lymphoma. Crit Rev Oncol Hematol.

[9] Sharma B, Pavelock N, Antoine M, Shah M, Galbraith K, Rawlins S (2019). Primary diffuse large B-cell lymphoma of the descending colon. Am J Med Sci.

[10] Sweetenham JW (2005). Diffuse large B-cell lymphoma: risk stratification and management of relapsed disease. Hematology Am Soc Hematol Educ Program.

[11] Mert D, Merdin A, Ceken S, Dal MS, Ertek M, Altuntas F (2021). Evaluation of hepatitis B virus, hepatitis C virus, and human immunodeficiency virus seroprevalence in patients with diffuse large B cell lymphoma and Hodgkin's lymphoma. J Cancer Res Ther.

[12] Tilly H, Gomes da Silva M, Vitolo U (2015). Diffuse large B-cell lymphoma (DLBCL): ESMO Clinical Practice Guidelines for diagnosis, treatment and follow-up. Ann Oncol.

[13] Carbone PP, Kaplan HS, Musshoff K, Smithers DW, Tubiana M (1971). Report of the committee on Hodgkin's disease staging classification. Cancer Res.

[14] Candelaria M, Dueñas-Gonzalez A (2021). Rituximab in combination with cyclophosphamide, doxorubicin, vincristine, and prednisone (R-CHOP) in diffuse large B-cell lymphoma. Ther Adv Hematol.

